# CAREGIVER WELL-BEING OUTCOMES IN A STROKE TRANSITIONS TRIAL: THE EFFECTS OF CAREGIVER BURDEN AND PREPARATION

**DOI:** 10.1093/geroni/igad104.1513

**Published:** 2023-12-21

**Authors:** Amanda Woodward

**Affiliations:** Michigan State University, East Lansing, Michigan, United States

## Abstract

The Michigan Stroke Transitions Trial (MISTT) was an open, pragmatic randomized trial which tested the effectiveness of a 60-day in-home social work case management for patients and caregivers transitioning home after hospitalization for a stroke. This secondary analysis explores the effect of caregiver burden and preparation on caregiver outcomes. The analytic sample is the 169 caregivers who participated in MISTT. Dependent variables are the Bakas Caregiver Outcomes Scale (BCOS) and the Patient Health Questionnaire-9 (PHQ9) at 90 days after discharge home. Key predictors include the Preparedness for Caregiving Scale and the Oberst Caregiving Burden Scale which measures caregivers’ perception of burden in relation to the both the time and difficulty of caregiving tasks. Analyses that controlled for outcomes at 7 days post discharge as well as caregiver age, marital status, and education were conducted using Stata v.16. Results indicate that caregivers who perceived caregiving tasks as more difficult had worse outcomes as measured by the BCOS. In addition, there was a significant interaction between task difficulty and age such that the effect of task difficulty on BCOS declined with age. In addition, depression was associated with greater task difficulty and less preparation. This study contributes to a growing body of work related to the complexity of transitions of care and has implications for how we support caregivers as their care recipient moves from inpatient to outpatient care.

